# Knowledge, attitude and practices of medical and health science students on the antiretroviral based HIV post-exposure prophylaxis in an Ethiopian hospital: an institutional based cross-sectional study

**DOI:** 10.1186/s12913-019-4611-2

**Published:** 2019-10-21

**Authors:** Betelhem Anteneh, Sewunet Admasu Belachew, Alem Endeshaw, Zewdu Birhanu Wubneh, Barun Ranjan Sarkar

**Affiliations:** 1 Department of Pharmacognosy, School of Pharmacy Gondar, University of Gondar-College of Medicine and Health Sciences, Gondar, Ethiopia; 2 Department of Clinical pharmacy, School of Pharmacy Gondar, University of Gondar-College of Medicine and Health Sciences, Gondar, Ethiopia; 3 Department of Pharmaceutics and Social Pharmacy, School of Pharmacy Gondar, University of Gondar-College of Medicine and Health Sciences, Gondar, Ethiopia; 4 Department of Pharmacology, School of Pharmacy Gondar, University of Gondar-College of Medicine and Health Sciences, Gondar, Ethiopia

**Keywords:** Post exposure prophylaxis, KAP, HIV/AIDS, Healthcare students, Gondar, Ethiopia

## Abstract

**Background:**

HIV/AIDS in resource-limited settings poses a high risk of occupational exposure to healthcare workers due to higher number of HIV infected patients. Hence, antiretroviral based post-exposure prophylaxis (PEP) for HIV is very crucial. The aim of the study was to determine the knowledge, attitudes, and practices of medical and Health science students on antiretroviral based HIV PEP in University of Gondar comprehensive specialized hospital (UOGCSH), Northwestern Ethiopia.

**Methods:**

An institutional-based cross-sectional study was conducted among 220 medical and health science graduating students in UOGCSH from May to July 2015. Data were collected using a self-administered pretested questionnaire. The collected data were analyzed using SPSS software version 22. Results were summarized in frequencies, percentages, and means with standard deviations and presented using tables or figures.

**Results:**

Among the respondents, only sixty-six (30%) of the 220 study participants has had adequate knowledge about HIV PEP. Furthermore, over 90 % of the students had positive attitude towards HIV PEP f. Out of the total respondents, 37/220 (16.8%) were in need of HIV PEP and of these students only 18/37 (48.6%) took PEP. On the other hand, merely 50% of the study subjects completed the full course of HIV PEP, while the rest 50% failed to finish. As to the respondents self-report, the sole reason for starting but failing to complete the full course of HIV PEP was intolerance to the side effects of antiretroviral.

**Conclusions:**

Although majority of the respondents had poor knowledge and practice, they owned a good attitude towards HIV PEP. Therefore, a pre-service intensive training for all students regarding HIV PEP prior to their clinical attachments is mandatory. In addition, potential side effects of ARTs and its managements should be priory informed to the students so as to prevent the associated non-adherence to ultimately reduce the incidence of drug resistance. Moreover, the habit of needle stick injury reporting was found to be poor that needs due improvement and there has been also a pressing need to supply sufficient protective barriers to the students while planning and rendering services.

## Background

HIV/AIDS is a leading cause of death in sub-Saharan Africa (SSA) with at least two-third of the world’s HIV infected people living in this region [1]. Ethiopia is among the SSA countries with approximately 800,000 people living with HIV/AIDS. Although antiretroviral therapy (ART) has significantly improved the management and prevention of HIV, early initiation of post-exposure prophylaxis (PEP) reduces the likelihood of sero-conversion of high risk exposure to HIV. A study showed that early administration of a short course of ART (as PEP for 28 days) reduces the risk of HIV transmission by 81% [2].

Healthcare provision, especially in resource-limited settings with profound infectious diseases burden including HIV/AIDS poses a high risk of occupational exposure to Healthcare Workers (HCWs) via different routes. For instance, percutaneous injuries like needle-stick or other sharp injuries, human infected blood, muco-cutaneous injuries (splash of blood or other infective body fluids) or other contacts [3, 4]. As a result of the high prevalence of communicable diseases, HCWs are often exposed to potentially infectious pathogens and increased occupational risk of these pathogens like HIV, Hepatitis B and C virus [5]. The World Health Organization (WHO) estimated 90% of the occupational risk to HIV occurs in healthcare settings of developing countries [6]. In the context of HIV, PEP refers to the set of services that comprises first aid, counselling, risk assessment, relevant laboratory investigations, following short course of PEP for 28 days and monitoring [5]. Since the early 1990s, antiretroviral medicines such as Tenofovir with emtricitabine and protease inhibitors, or Zidovudine with lamivudine and protease inhibitors as alternative regimens were recommended as standard PEP for occupational exposure to HIV [7].

Indeed, studies stated that there is an information gap in the healthcare setups regarding HIV PEP. For instance, a study conducted in Iran indicated that only half of the nursing and midwifery students had good awareness about HIV PEP [8]. Similarly, a study conducted in Ethiopia, Hawassa University showed that one-third of the midwifery and nursing students were exposed to HIV risk factors and among those only 59.3% took PEP [9].

In addition, a study conducted in an Indian medical college demonstrated merely 37.6% had sufficient knowledge regarding HIV PEP and an even smaller number 20.7% had information about the right time of initiation of HIV PEP [10]. Moreover, a Serbian study mentioned that 87% of the HCWs had not been informed about the guidelines regarding protection from HIV, and reported that PEP protocols were not available at their work places [11]. In line with this, 87.5% of the nursing students in Zambia were unaware of PEP services [12]. Evidence from those studies suggests that healthcare students are at high risk of occupational exposures. With this, it is important for healthcare students to have sufficient knowledge to protect themselves while they are in service oriented clinical trainings in healthcare institutions. Furthermore, the important factors that influence the overall risk for occupational exposures to blood borne pathogens include the number of infected individuals in the patient population [13] and this has strengthen the point that in Ethiopia the overall risk for occupational exposure to HIV is high since the disease burden is high in the nation [2].. In addition, a study done in Gondar which is the current study area found quite high prevalence of health care workers exposure to HIV/AIDS risk factors in the health care settings in Gondar city [3]. In addition, in the current set up the existing strategy to prevent occupational HIV infection is through wearing protection gloves. However, some students forgot wearing the protection at all time. A 1 month triple therapy comprised of TDF + 3TC + EFV/NVP/LPV/r as PEP has been also used for the students who are already exposed to the infection. In this regard, there is no information about graduating students’ awareness towards HIV PEP in general although it is remained to be crucial to be informed of it particularly for those early practitioners.

Furthermore, to the best of the authors’ knowledge and search, no previous study explored healthcare students’ knowledge, attitude and practice towards PEP for HIV in the current study area. Thus, this study was aimed to assess the knowledge, attitudes, and practices towards ART based HIV post exposure prophylaxis among medical and health science graduating students who were providing clinical services while practicing on real patients in UOGCSH, Gondar, Northwestern Ethiopia.

## Methods

An institutional-based cross-sectional study was conducted among Medicine and Health Sciences (MHS) graduating students of Gondar College of medicine and health sciences, University of Gondar from May to July 2015. UOGCSH is a referral hospital with a huge number of beds, serving more than 7 million people living in and around Gondar town. Gondar is found in Amhara region of Ethiopia. According to Demographics and Health survey (DHS) 2005 report, the prevalence of HIV/AIDs in Amhara region was found to be 1.3% in the population. However, the prevalence of HIV/AIDs in Gondar population has not been studied. Instead the prevalence of HIV with visceral leshimaniasis co-infection has been reported to be 17.75% [14].

Generally, since most of the tasks of the MHS graduate class students are potentially risky to the students compared to the previous exposures, great attention and preparation are required to avoid acquiring HIV infection.

### Sample size determination and sampling technique

**Fig. 1:**
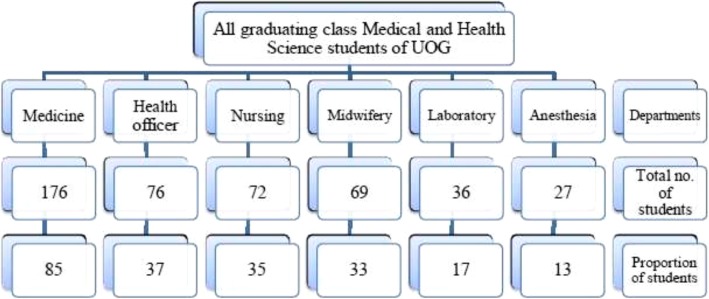
Flow chart showing sampling of study participants

The sample size was determined by employing a single proportion formula (n = [Z α / 2] 2 P (1-p] / d2) at 95% confidence interval, where, Z α / 2 = 1.96, P = prevalence of 50% was taken since there was no similar previous study in the study area and d = 5% of marginal. With this calculation, we obtained 384 + 38 (10% dropouts) =422 as sample size. Since the exact number of respondent population is less than 10,000, we used correction formula of nf = ni/(1 + ni/N), where nf = calculated sample size, ni = reduced sample size, and N = total number of all the source population. Therefore, (422/1 + 422/456) given a final sample size of 220. The final sample size was drawn by employing a stratified random sampling technique. With this, the departments were identified to be classified in to 6 strata. After that, the number of students in each stratum (department) was determined and this was used to proportionally allocate the calculated sample size for each departments. In the 6 departments, 456 students were identified and the six departments had different number of students. With this, to proportionally allocate the sample size for each departments based on their respective number of students, we have calculated the proportion as follows: D = n/456*220. Where, “n” is the number of the students in each departments and “D” is a final determined sample size for each department. “D” then finally was drawn from “n” by employing a simple random sampling technique via lottery method (Fig. 1).

### Data collection tool and quality control technique

The questionnaire was comprised of 31 items and made to have four sections that constitutes 5 items related to socio-demographic characteristics, seventeen questions related to knowledge statements about HIV PEP with “Yes/No” or “Correct/Incorrect” responses. Of these, two questions were related to source of information and previous training experiences with multiple options to select at least one of three choices. A five statement questionnaire containing “Yes/No” were used to assess the attitudes and four questions for participants’ practice towards HIV PEP. Be informed that, this structured self-administered questionnaire containing socio-demographic characteristics and questions that can assess the level of knowledge, attitude, and practice towards PEP for HIV was adopted from previous literatures [5–12] and modified to suit to our study set up. The tool was prepared in English language only and pretested among 15 % of the total sample size which was not included in the final analysis.

### Data collection and management

The developed data collection tool was provided to study subjects by a six trained data collectors. The data collectors explained the objective of the study for the participants before they let them fill the questionnaire. Further, the data collectors mentioned as they clarified any ambiguous and unclear questions when necessary.

### Data analysis

Data were entered, cleaned, and coded for analysis using SPSS software version 22 for Windows. Results were summarized in frequencies, percentages, and means with standard deviations f and presented using tables or figures. In the current study, operational definitions has been included to clarify core terms. With this, respondents’ level of knowledge and attitude was measured by calculating the total possible correct scores in PEP for HIV questions and classified into two categories:

- **Adequate knowledge:** Respondents answered more than 70% of the knowledge questions correctly.
- **Inadequate knowledge:** Those who scored less than 70% of the knowledge questions correctly.
- **Good attitude:** Respondents that scored 70% and above for five attitude statements.
- **Poor attitude:** Respondents scored < 70% on the given attitude questions.
- ***PEP practice*** reported as respondents’ having used post-exposure prophylaxis (PEP) of antiretroviral therapy (ART) medications for HIV following occupational exposure to HIV high-risk conditions.
- **Post-exposure prophylaxis (PEP)** is a short term antiretroviral treatment given to reduce the likelihood of HIV infection after potential exposure occupationally while treating HIV patients.

### Ethical clearance

The study protocol was approved by ethical review committee of School of Pharmacy, University of Gondar with an approval number of UoG-SoP-10/2015. The purpose of the study was briefly explained to the participants and asked whether they agree (give consent) to participate. It was an informed consent asked verbally and told tick “Yes” when agreed to participate in the study. Furthermore, confidentiality of the study subjects was maintained throughout the study period by not mentioning any personal identifier in the questionnaire and told that participation in this study is fully voluntarily so that they can withdraw anytime.

## Results

A total of 220 MHS graduating students were approached, given a response rate of 100%. The majority of the participants were males (62.7%) with a mean (SD) age of 23.43 ± 2.19 years. Almost all the respondents were single (96.4%) and nearly three-quarter were Orthodox Christians (72.3%). Moreover, the highest number of respondents were medical interns (38.6%) followed by comparable number of respondents from health officers (16.8%) and nursing students (15.9%) (Table 1).

**Table 1:** Socio-demographics of study respondents, UOGCSH, 2015, *N* = 220

Variables	Categories	N (%)
Age (Years)	20–22	76 (34.5)
	23–25	110 (50.0)
	> 25	34 (15.0)
Sex	Male	138 (62.7)
	Female	32 (37.3)
Marital status	Married	8 (3.6)
	Single	212 (96.4)
Religion	Orthodox Christian	159 (72.3)
	Protestant Christian	34 (15.5)
	Muslim	26 (11.8)
	Catholic Christian	1 (0.5)
Profession	Medicine	85 (38.6)
	Health Officer	37 (16.8)
	Nursing	35 (15.9)
	Midwifery	33 (15.0)
	Laboratory	17 (7.7)
	Anesthesia	13 (5.9)

### Knowledge level of MHS students about PEP for HIV

Only sixty-five (29.5%) of the study participants had adequate knowledge about HIV PEP. Almost all of the respondents (97%) were aware of HIV PEP., and students ranked class room lectures (50.9%) as the major source of information regarding HIV PEP, followed by formal training and seminars. Ninety-five study subjects knew the effectiveness of HIV PEP correctly. On the other hand, only 20 out of the 220 respondents (9.1%) correctly identified blood fluids as a high risk source for HIV transmission (Table 2).

**Table 2:** Response of graduate heath care students to each questions that assess their knowledge about PEP in UOGCSH, 2015, N = 220

Knowledge questions	Responses	Frequency (%)
Heard about PEP	Yes	215 (97.7)
	No	5 (2.3)
Know the use of PEP	Yes	208 (94.5)
	No	12 (5.5)
PEP reduces likelihood of HIV	Yes	202 (91.8)
	No	18 (8.2)
PEP prolongs life of person	Yes	17 (7.7)
	No	203 (92.3)
High risk of blood fluid	Correct	20 (9.1)
	Incorrect	200 (90.9)
Effectiveness of PEP	Correct	95 (43.2)
	Incorrect	125 (56.8)
Lack of Protective barriers is a perceived cause of HIV	Yes	139 (63.2)
	No	81 (36.8)
Work load is a perceived cause of HIV	Yes	80 (36.4)
	No	140 (63.6)
Lack of standard precautions is perceived cause of HIV	Yes	92 (41.8)
	No	128 (58.2)
PEP given in hospital	Yes	198 (90.0)
	No	22 (10.0)
Best time to start PEP (72 h)	Correct	166 (75.5)
	Incorrect	54 (24.5)
Consider PEP after cleaning the wound	Yes	176 (80)
	No	44 (20)
Attended training related PEP	Yes	90 (40.9)
	No	130 (59.1)
When did you received training	Before 1 year	64 (29.0)
	In past 1–2 years	18 (8.2)
	Before 3 years	6 (2.7)
Information about available PEP guidelines	Yes	90 (40.9)
	No	130 (59.1)
Mentioned at least two drugs given for PEP	Yes	76 (34.5)
	No	144 (65.5)
Source of PEP information	Classroom lectures	111 (50.5)
	Training	66 (30.0)
	Personal study	31 (14.1)
	Seminar	8 (3.6)

**Fig. 2:**
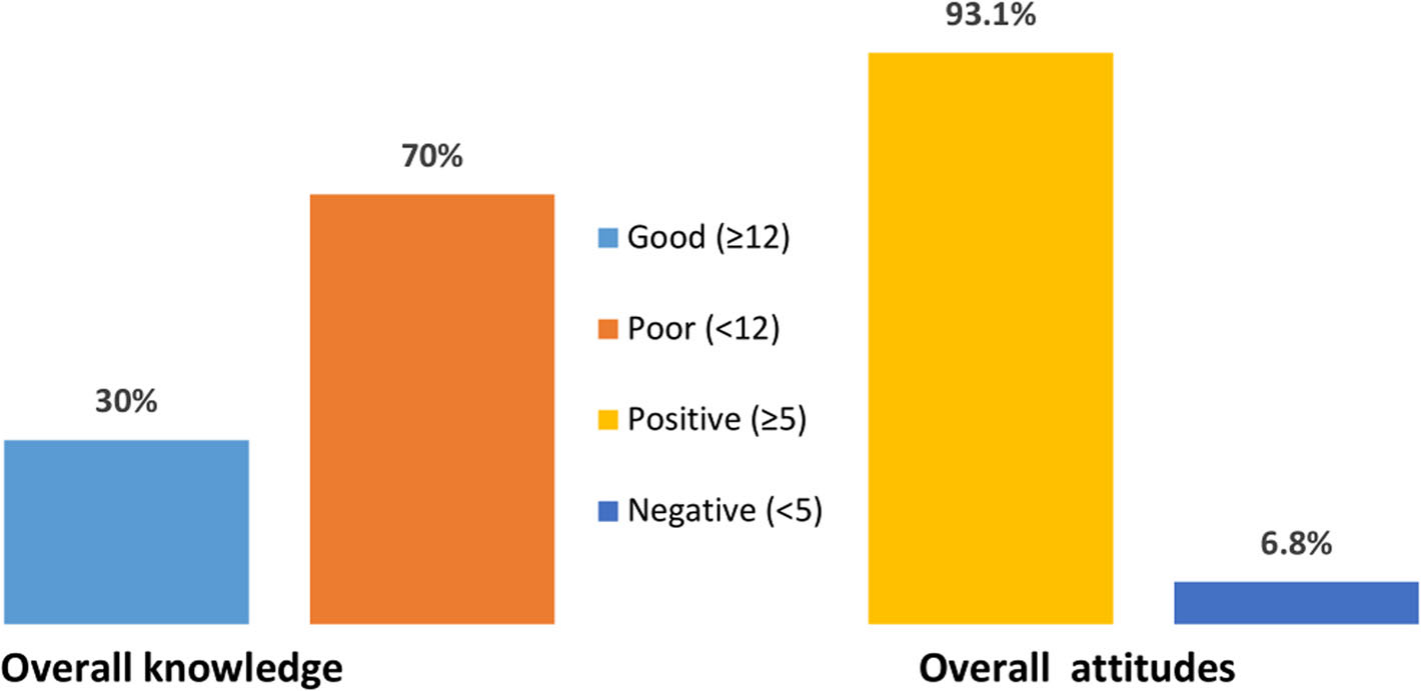
Mean scores of respondents’ knowledge and attitude towards HIV PEP

Nearly 60% of the participants were unaware of the availability of PEP guidelines, and only 46.8% of respondents claimed that needle stick injuries need to be reported. On the other hand, although three-quarter (75.5%) of study subjects knew the best time to initiate PEP regimens for HIV, only 30.5% were able to mention the drugs given for HIV PEP. Overall, 70% of the respondents showed poor knowledge towards HIV PEP (Fig. 2).

### Attitude of students about PEP for HIV

More than 90 % of the respondents agreed as HIV PEP is important and mentioned the presence of HIV PEP guidelines in the hospital or work place is mandatory. The beliefs of respondents whether HIV PEP can reduce the likelihood of HIV infection/disease progression once exposed to the virus were assessed. To this fact, almost all of the respondents (98.8%) strongly agreed that PEP can reduce the probability of HIV transmission. Nearly half of the students (46.8%) believed that reporting needle stick injuries remained to be imperative (Table 3). Overall, more than 90 % of the graduating MHS students had positive attitude towards HIV PEP (Fig. 2).

**Table 3:** Student respondents’ attitude about PEP in UOGCSH, 2015, N = 220

*Questions*	*Responses*	*Frequency (%)*
PEP is important	Yes	214 (97.3)
	No	6 (2.7)
There should be guidelines in working areas	Yes	209 (95.0)
	No	11 (5.0)
PEP reduces the likelihood of HIV transmission after occupational exposure	Yes	213 (98.8)
	No	7 (3.2)
Believe PEP works	Yes	216 (98.2)
	No	4 (1.8)
Reporting needle stick injuries is important	Yes	103 (46.8)
	No	117 (53.2)

### Practice status of MHS students towards PEP for HIV

Out of the total respondents, only 37/220 (16.8%) were in need of HIV PEP. However, merely 18/37 (48.6%) of them reported as they had HIV PEP. On the other hand, only 50% of the students who started PEP was able to complete the full course of HIV PEP while the rest 50% of the students already started HIV PEP but failed to finish the full course. In line with this, all of the students who terminated the HIV PEP mentioned the side effects of antiretroviral drugs as their main reason for discontinuation. (Table 4).

**Table 4:** Healthcare students Practice of PEP for HIV in UOGCSH, 2015, N = 220

Questions	Responses	Frequency (%)
Ever exposed to HIV (needing PEP)	Yes	37 (16.8)
	No	183 (83.2)
Placed on PEP	Yes	18 (8.2)
	No	202 (91.8)
Completed the PEP course	Yes	9 (50)
	No	9 (50)
Reasons for discontinuation	Side effects of drugs	9 (50)

## Discussion

In the present study, the KAP of graduating MHS students on ART based HIV PEP were assessed. The main reason for considering final year graduating students in this study was because they are known to actively involve in patient care at the institutional hospital. In this regard, those students are usually at a greater risk for acquiring blood-borne infections in the workplace as they involve in direct patient care. The paucity of published data on HIV PEP among MHS graduating students in Ethiopia motivated the conception of the current study that aimed to assess the knowledge, attitudes and practices of MHS graduating students on ART based HIV PEP.

In this study, almost all the MHS students had heard of ART based HIV PEP and in this regard, medical students took the lion share. However, none of the laboratory and anesthesia students know anything regarding HIV PEP. In comparison, the current study findings showed higher level of awareness than a study conducted in Hawassa University (67.1%) students [9]. The actual reasons for these differences were not clearly known, but it may be attributed to various awareness levels of experiences in their workplace. Interestingly, nearly 70 % of the medical students in our study got information related to HIV PEP from their medical training.

Nearly, three-fourth (75.5%) of the respondents were aware of the time when PEP is needed to be initiated and correctly reported that it should be done within 2 hours from the instance of HIV exposure. In this regard, the percentage of awareness among participants in this study were higher compared to the result in Hawassa university midwifery and nursing graduating students (48.1%) [9], but much lower than Nigerian health professionals (97%) [15] and Cameroon nurses (83.5%) [15].

Overall, 70 % of MHS graduating students had poor knowledge score on HIV PEP. Comparable results were also noticed among health care workers in Cameroon (73.7%) and Zimbabwe (65%) [16, 17]. Incomparably, higher level of fair and adequate knowledge was noticed among nurses in Nepal (68%) and UOGCSH health professionals (63.1%) in Ethiopia [5, 18].

Indeed, the knowledge on risky body fluids that can potentially transmit HIV was found to be quite poor among the present study population, with more than 90 % of the respondents being unable to identify potentially high risk body fluids. In line with this, only 9.1% correctly identified cerebrospinal fluid as a high risk body fluid for HIV transmission. To the contrary, a multicenter study done in eastern part of Ethiopia stated that very high percentage (97.9%) of healthcare workers mentioned that blood and various body fluids can transmit HIV/AIDS and even claimed that they can correctly identify body fluids that can potentially transmit different diseases. This might be due to their longer experience as the health professional (mean, 8.2 years) that render them learn such health events from their lengthier experiences unlike the current study in which the professionals were still on basic trainings building up experiences [19]. Unlike, the findings of the current study, a study conducted among nurses in Cameron (< 50%) and Nepal (65%) noted as the professionals correctly identified the high risk fluids for HIV transmission [16, 18]. Similarly, high rates of correct identification of risky body fluids were also revealed a study done among Nigerian family physicians revealed that the doctors clearly identified the risky body fluids [20].

On the other hand, less than a 50 % of our study participants believed that HIV PEP is effective in preventing HIV transmission. The major perceived cause of exposure to HIV risk in UOGCSH was lack of protective barrier followed by lack of standard precautions, and work load. Likewise, similar barriers were also reported in other studies done among nurses and midwifery students in Addis Ababa as well as among health workers in southwest Ethiopia, and Malaysia hospitals [5, 21, 22].

Regarding appropriate time for initiation of HIV PEP, three-fourth of the respondents knew how soon to initiate HIV PEP for exposed individual following needle stick injury. In comparison, these findings were much higher than the studies conducted among dental interns and postgraduate dental students in India (20.4% versus 42.2%) [23], Mulago hospital workers in Uganda (22.3%) [24], Gondar health workers in Ethiopia (50.8%) [5], Nepal (60%) [18], Mumbai (64%) [25], and Cameron (66%) [16]. These differences in their knowledge could be attributed to the difference in overall knowledge imparted during their academic training as well as to discrepancies’ in study populations and health care settings.

The current study also uncovered that more than 90 % of MHS graduating students exhibited positive attitude (93.1%) towards HIV PEP. It can be stated that a positive attitude was noticed in almost all the students from midwifery, nurses, medicine, laboratory, health officers and anesthesia. For example, more than 95 % of our respondents revealed as ART based HIV PEP is important and availability of PEP guidelines in the hospital or in their work place is unquestionably crucial. Comparably, our results were much higher than a study conducted in UOGCSH health workers where 88.5% agreed on the need of PEP guidelines in work areas and nearly 70 % believed that PEP can be effective for preventing HIV transmission.

Even though needle stick or short objective injuries are one of the important sources of HIV transmission, only 43.8% of the respondents believed that reporting such injuries was important. Generally, to comment the actual risk of transmission of HIV or hepatitis up on exposure to risky conditions: In our set up, professionals usually get exposure to HIV/Hepatitis while they are shouldering their day to day activities in the hospital as a result of professionals negligence to protect themselves all the time because of their super busy duty. Years later, when professionals are asked on how they got HIV/Hepatitis, they explained that they had exposure to needle injury, blood splash on eye etc. when they provide care to a known HIV/Hepatitis patient. Considering such frequent cases, it can be concluded that the actual transmission have been always true after exposure and is alarmingly high in our set up.

In our study, 37 (16.8%) respondents were in need of PEP for HIV, but only 18 (8.1%) received PEP and 50% of the respondents who started PEP were able to complete 28 days of the PEP regimen. However, the remaining 50% of the students who started PEP failed to complete the full course of PEP. These findings were much higher than a Nigerian study in Abuja teaching hospital where only 6% of exposed participants received PEP [26]. Indeed, our results were in line with other studies conducted in Ethiopia, Cameron, Uganda, Kenya and Tanzania [5, 16, 23, 26, 27]. Furthermore, a study conducted in Gujarat state, India mentioned that a higher number (> 95%) of participants were able to complete the regimen [28]. This might be due to the fact that in the India study the participants had already completed their training and were employed professionals so that they would have better experience to PEP use than the current study in which the participants are still on training that definitely lack experience. In addition, the side effect profile of PEP initiated in the Indian study was minimal with no life threating adverse reaction described so that this further promotes the completion of the prophylaxis. Generally, completion of the initiated PEP is crucial for a better prevention and in our study PEP completion issues still needs effort to be improved.

Previous studies conducted on PEP for HIV in different countries highlighted the importance of formal training on PEP for HIV and that awareness of availability of PEP guidelines in hospitals were significantly associated with good knowledge of PEP. Contradictorily, our study participants received classroom lectures (50.5%), trainings (30%) and personal study (14.1%) but failed to demonstrate adequate knowledge of PEP.

### Study limitations

Although the study is the first in its kind in the study area that the findings could be used as an input to improve the antiviral based PEP service, the study has not been left without limitations. Firstly, the study was a cross-sectional study conducted in a single institution in Ethiopia and the results of our findings cannot be generalized to other hospitals in Ethiopia. In addition, the results of the present study highly depends on honesty and faith of the respondents as the mere data collection method used was using self-administered questionnaire.

## Conclusion

Poor knowledge and practices towards PEP regimen were noticed among medical and health science students, but they had a good attitude towards PEP. To this end, a formal pre-service training for all students regarding PEP for HIV prior to their clinical attachments is of utmost importance. The training should include a brief explanation regarding the bad consequences of non-adherence that primarily include poor treatment outcome and greatest risk of resistance to the only and few donated ART regimens available in Ethiopia to be used for both treatment and prophylaxis of HIV/AIDS. Likewise, as a main driver for non-adherence to PEP, common temporal side-effects of PEP and its managements should be priory informed to healthcare workers to reduce its impact on adherence. Furthermore, the habit of reporting needle stick injury was poor that needs efforts to improve. Most importantly, the effort will be through promoting the culture of reporting and by making the reporting process easy. In fact, reporting such injuries as early as possible will teach others not to commit same problem again and generally, will enable stake holders intervene the issue before it re-occur. Finally, as far as PEP use has left with so many inconveniences, risk reduction is the paramount choice. With this, taking the heightened importance of protective barriers so as to reduce the risk of exposure to HIV, different stakeholders (the hospital equipment supply chain manager, the HIV/AIDS clinic, the hospital director, equipment and supply quality assurance team and other administrative bodies) should work together in co-ordination to secure the supply and assure the quality of those vital protective barriers and to further insist health workers to always wear protective barriers when exposed to HIV risk factors as well as to dispose properly once done. Finally, we recommend future investigators to conduct a prospective multicenter studies with additional objectives (including predictor analysis) for better generalization and outcome.

## Data Availability

The datasets used and/or analyzed during the current study available from the corresponding author on reasonable request.

## Abbreviations

3TC: Lamivudine
ART: Antiretroviral Therapy
DHS: Demographics and Health survey
EFV: Efavirenz
HCWs: Health Care Workers
HIV: Human Immunodeficiency Virus
KAP: Knowledge, attitude and practice
LPV/r: Lopinavir/ritonavir
MHS: Medical and health sciences
NVP: Nevirapine
PEP: Post-exposure, prophylaxis
SOP: School of Pharmacy
SPSS: Statistical package software for social sciences
SSA: Sub-Saharan Africa
TDF: Tenofovir disoproxil fumarate
UOG: University of Gondar
UOGCSH: University of Gondar Comprehensive Specialized Hospital
